# Salivary Immunoglobulin A Secretion Rate Is Negatively Associated with Cancer Mortality: The West of Scotland Twenty-07 Study

**DOI:** 10.1371/journal.pone.0145083

**Published:** 2015-12-23

**Authors:** Anna C. Phillips, Douglas Carroll, Mark T. Drayson, Geoff Der

**Affiliations:** 1 School of Sport, Exercise & Rehabilitation Sciences, University of Birmingham, Birmingham, England; 2 Clinical Immunology Service, College of Medicine and Dentistry, University of Birmingham, Birmingham, England; 3 MRC/CSO Social and Public Health Sciences Unit, University of Glasgow, Glasgow, Scotland; University of the Balearic Islands, SPAIN

## Abstract

Immunoglobulins are essential for combating infectious disease although very high levels can indicate underlying pathology. The present study examined associations between secretory immunoglobulin A (sIgA) in saliva and mortality rates in the general population. Participants were 639 adults from the eldest cohort of the West of Scotland Twenty-07 Study aged 63 years at the time of saliva sampling in 1995. From unstimulated 2-minute saliva samples, saliva volume and S-IgA concentration were measured, and S-IgA secretion rate determined as their product. Mortality data were tracked for 19 years. Cox proportional hazard models were applied to compute hazard ratios (HR) for all-cause mortality from sIgA secretion rate. Associations were adjusted for gender, assay batch, household occupational group, smoking, medication usage, and self-reported health. There was a negative association between log sIgA secretion rate and all-cause mortality, HR = 0.81, 95%CI = 0.73–0.91, *p* < .001. Further analysis of specific causes of mortality revealed that the all-cause association was due to an underlying association with cancer mortality and in particular with cancers other than lung cancer. The HR for non-lung cancer was 0.68 (95%CI = 0.54 to 0.85) implying a 32% reduction in mortality risk per standard deviation rise in log sIgA secretion rate. Effects were stronger for men than women. For deaths from respiratory diseases, sIgA secretion had a non-linear relationship with mortality risk whereby only the very lowest levels of secretion were associated with elevated risk. SIgA concentration revealed a similar but weaker pattern of association. In the present study, higher secretion rates of sIgA were associated with a decreased risk of death from cancer, specifically non-lung cancer, as well as from respiratory disease. Thus, it appears that sIgA plays a protective role among older adults, and could serve as a marker of mortality risk, specifically cancer mortality.

## Introduction

Immunoglobulins (Ig) or antibodies are proteins secreted by white blood cells (B lymphocytes) which circulate in the body and tag, destroy, and/or neutralize bacteria, viruses, and other harmful or foreign materials (antigens). This is achieved by opsonising or coating foreign materials which marks them for destruction or neutralization [1]. Secretory IgA (sIgA) is secreted at the mucosal surfaces (e.g., mouth, nose, gastrointestinal tract) [2] and can be measured in saliva. SIgA is the first line of defence against infection at these surfaces, acting to prevent colonization by microbes [3, 4]. It is considered particularly key in the defence against viral and bacterial infections of the upper respiratory tract (URTIs), such as colds and influenza [5]. However, the relationship between sIgA and health is complex and subject both to confounding and reverse causation. For example, in the case of oral health, lower levels of sIgA have been shown to be a risk marker for dental caries and decay [6] but high levels have been deemed an indicator of current oral infection [7–9].

Salivary IgA has previously been shown to be a stress marker in humans. For example, we have previously shown that low levels of sIgA are associated with caregiving stress in older age [10], higher ratings of the stressfulness and disruption caused by negative life events [9, 11–15]. Low sIgA is thought to be an important underlying mechanism linking chronic stress with URTIs [16] and increased infections risk in some populations such as diabetic patients [17]. However, high levels of circulating immunoglobulins are also associated with disease. For example, higher IgA production in the bowel may also be part of the cause of inflammatory bowel disease [18]. Certain types of kidney disease are also associated with abnormalities of the IgA system [19]. Recently, in a large study of Vietnam-era war veterans, we have found that higher levels of serum immunoglobulins, including IgA, were associated with around a two-fold increased risk of mortality from all-causes and ‘other’ causes (corresponding to deaths that were not ascribed to cardiovascular disease and cancer causes, largely comprising infectious diseases) [20]. On the other hand, severe serum IgA insufficiency which is inherited by up to 0.5% of the general population is also associated with higher mortality in the first 10–15 years from diagnosis in a Swedish population study [21] and has also been related to higher prevalence of coeliac disease, type I diabetes and other autoimmune diseases [22]. Taken together, these findings present an interesting paradox regarding the utility of IgA as a marker of disease risk. Few studies have examined the associations between serum IgA and mortality, other than those above, or have focused on particular infectious disease states. Similarly, studies of salivary IgA have concentrated on IgA specific to particular pathogens, or in the context of specific disease states. To our knowledge, no studies have examined the prospective associations between salivary IgA and mortality in the general population. Consequently, in the present analysis of data from the West of Scotland Twenty-07 Study on a large older adult community sample, we examined the associations between sIgA secretion rate and mortality, adjusting for a range of covariates that might be considered confounders (e.g. socio-economic status, gender, smoking etc.) due to their known associations with mortality and/or IgA level. Although we have examined health and behavioural associations with sIgA and mortality independently in this cohort, this new analysis is the first examination of potential associations between sIgA and mortality.

## Materials and Methods

### Participants

Data were derived from the oldest of three age cohorts in the West of Scotland Twenty-07 Study [23, 24]. These data are freely available from the West of Scotland Twenty-07 Study in accordance with the Medical Research Council UK’s guidance on data sharing at http://2007study.sphsu.mrc.ac.uk/Information-on-data-sharing.html. Participants were sampled from 52 postcode sectors in the Central Clydeside Conurbation, a predominantly urban area centred on the City of Glasgow, using the electoral register enhanced to list the age and sex of all residents in the household. Postcode sectors were chosen to reflect a spread of social advantage and disadvantage. Participants were chosen randomly with probability proportional to the overall population of the same age within a postal code area [25]. Participants were initially recruited in 1988/89 (wave 1). They were followed up on four subsequent occasions over the following 20 years. The Twenty-07 Study’s principal aim was to investigate the processes that generate and maintain socio-demographic variations in health [26]. Three narrow age cohorts were chosen, each reflecting important stages of life and transitions. The oldest cohort was selected to reflect the transitions from middle to old age and from work to retirement; only the oldest cohort is considered in this analysis, thus inclusion criteria were aged 55 years in 1987 living in the selected area and available in 1995 for saliva collection.

A comparison of the cohorts with equivalent samples drawn from the 1991 UK census indicated homogeneity in terms of sex, occupational group, and home ownership [27]. The sample was almost entirely Caucasian, reflecting the West of Scotland population from which it was drawn. Demographic and health-related data, such as smoking, body mass index, and blood pressure, were collected at each wave. Vital status was continuously monitored. Wave 3, conducted in 1995 to 1996, forms the baseline for this analysis as samples of saliva were collected at that wave in order to investigate variations in sIgA. National Health Service or University of Glasgow ethics committee approval was obtained for each wave of the study and all participants provided written informed consent, and were free to withdraw at any time.

### Procedure

Participants were interviewed in their own homes by nurses who were trained in a standardised manner in how to administer a standardised interview schedule and collect the saliva sample. Household occupational group was classified as manual and non-manual from the occupational status of the head of household, using the Registrar General’s Classification of Occupations [28]. For those who had retired their occupation prior to retirement was used. Smoking behaviour was determined by responses to the question, ‘Do you ever smoke tobacco now? I am thinking of a pipe, cigars and your own roll ups as well as cigarettes you might buy’. Participants were asked to indicate their level of health in response to the prompt: ‘would you say that for someone your age your own health is…’ with the response options: excellent, good, fair, or poor. For the present analyses, self-reported health was dichotomised into excellent and good versus fair or poor as very few participants endorsed excellent or poor. The number of medications used was ascertained from several questions asked throughout the interview. It was dichotomised at 4 and is included in the analysis to allow for the effect on saliva secretion. The study participants were flagged at the UK’s National Health Service Central Registry, which provided notifications of death and cause.

Saliva samples were taken at the end of the interview but before taking any other physical measures. We used a standard salivette (Sarstedt Ltd., Leicester, UK). Respondents were instructed to swallow hard to dry out their mouth and then immediately to place the swab in their mouth under the tongue. They were asked to hold the swab as still as possible for 2 min. Nurse interviewers timed 2 minutes using a portable digital timer. After exactly 2 minutes, respondents removed the swab and returned it to the salivette case. All samples were frozen within 2 h of collection and remained frozen at -20°C until assay. Samples were recovered after thawing by centrifugation at 1000 g for 10 min. Secretory IgA concentration was measured by double antibody sandwich ELISA, described in detail elsewhere [29]. Intra-assay %CV was 3.8 and inter-assay %CV was 7.6. Prior to assay saliva volume was determined gravimetrically. Saliva sample collection and volume determination for sIgA analysis follows the procedure previously described [30]. The focus of analysis was S-IgA secretion rates (μg/2min), which were calculated as the product of saliva volume (ml) and S-IgA concentration (μg/ml).

### Data analyses

Cox’s regression models were used to analyse mortality risk both for all-causes and specific causes. Log sIgA was the primary predictor and analyses are presented controlling for key covariates: sex, household occupational group, smoking status, self-reported health and number of medications. Given that earlier studies have examined sIgA concentration, we have also conducted analogous analyses for sIgA concentration and mortality. Specific causes of mortality analysed were: cardiovascular disease (CVD), cancer, lung cancer, other cancers, respiratory disease, and other causes of mortality. There were too few cases of infectious disease (N = 2), other sub-categories of cancer or other causes of mortality to analyse. As previous studies have shown increased mortality risk for both high and low levels of sIgA, we also fitted models with polynomial terms in sIgA up to the fourth power. Fractional polynomials were used to display any significant non-linear relationships. Analysis was undertaken using SAS version 9.4 with the exception of the fractional polynomials which employed the coxphw package within R.

## Results and Discussion

Of the 1,042 participants who took part in the first wave of the Twenty-07 study, 723 were re-interviewed at the third wave–the baseline for the analysis here; 91 participants died before wave 3 leaving 951 participants eligible for participation. Those who did and did not take part in wave 3 were not significantly different in sex, smoking status or subsequent survival. They did, however, differ in terms of social class, with an 83% response rate in non-manual social classes compared with 71% in the manual social classes. Thirty-five did not provide a saliva sample and a further 49 had too little saliva to assay sIgA; thus, the analysis presented here is based on the 639 participants with complete data. Those with incomplete data were significantly different from the analysed sample in a number of ways: more were female (71% versus 53%); fewer had good self-reported health (44% versus 59%) and they were around two months older. Mean (SD) ages in the analysed and incomplete samples were 63.7 (0.7) and 63.6 (0.6) years, respectively. The groups were not significantly different in household occupational group, smoking, or vital status. Of the 639 in the analysed sample, 300 (47%) had died during the follow up period compared with 44 (52%) among those with incomplete data. The last death was recorded on 30^th^ May 2015 and the surviving sample was censored at that date.

### Treatment of sIgA raw data

Some of the early reports on sIgA were criticised for analysing concentration rather than secretion rate. Since concentration is partly determined by salivary flow, all results reported here take the total volume of saliva collected over the two-minute period and secretion rates (ugs/min) were calculated by multiplying concentration (ugs/ml) by total volume of saliva (mls) and dividing by two. Secretion rates not only control for the concentrating or diluting effects of salivary flow, they also, in conjunction with salivary flow, indicate better the actual protection offered by sIgA in efficiently coating the mucosa [31]. The distribution of sIgA is typically highly positively skewed, and was so in this sample with a skewness of 5.8. A log transformation reduced this to -0.47.

Table 1 gives descriptive statistics for log sIgA secretion rates, log concentration, salivary volume and vital status by demographic and control variables. There were sex differences for each of the saliva and sIgA measures and smokers had significantly lower levels of sIgA concentration and secretion than non-smokers. All control variables were significantly associated with mortality during follow up.

**Table 1:** Descriptive statistics for log sIgA concentration and secretion rates, salivary volume and vital status by demographic and control variables.

		N	Log sIgA concentration (μg/ml)		Log sIgA secretion rate (μg/2 min)		Saliva volume (ml)		Deceased	
			Mean (SD)/N(%)	*p*	Mean (SD)/N(%)	*p*	Mean (SD)/N(%)	*p*	(%)	*p*
**Female:**	Yes	340	4.34 (1.02)	.015	3.07 (1.69)	< .001	0.51 (0.49)	< .001	41	.003
	No	299	4.54 (1.06)		3.63 (1.60)		0.66 (0.55)		53	
**Smoker:**	Yes	207	4.21 (1.09)	< .001	3.10 (1.78)	.018	0.57 (0.51)	.620	63	< .001
	No	432	4.53 (1.00)		3.44 (1.60)		0.59 (0.53)		38	
**Taking 4+ medications:**	Yes	209	4.44 (1.04)	.855	3.21 (1.64)	.226	0.53 (0.49)	.074	59	< .001
	No	430	4.42 (1.04)		3.38 (1.68)		0.61 (0.54)		40	
**Good health:**	Yes	377	4.48 (0.97)	.161	3.43 (1.59)	.060	0.61 (0.53)	.088	37	< .001
	No	262	4.36 (1.14)		3.18 (1.77)		0.55 (0.51)		60	
**Manual Social Class:**	Yes	354	4.41 (1.06)	.569	3.27 (1.70)	.302	0.57 (0.51)	.387	54	< .001
	No	285	4.46 (1.02)		3.40 (1.63)		0.61 (0.54)		37	

Table 2 shows the Cox’s regression models with and without covariate adjustment for all causes and specific causes. Log sIgA secretion rate significantly predicted all-cause mortality such that one standard deviation increase in log sIgA was associated with a 19% decreased risk of death. This remained significant following adjustment for sex, assay batch, smoking status, medication usage, self-reported health and household occupational group, with individuals with higher secretions rates showing a 14% decreased mortality risk. The results for cause specific mortality show that the relationship is strongest for cancer and cancers other than lung cancer in particular. In the case of non-lung cancer, one standard deviation increase in log sIgA is associated with a 32% decrease in mortality risk and this effect is completely unaffected by adjustment for potential confounders. When death due to causes other than non-lung cancer were modelled, the effect of log sIgA is much weaker and non-significant (HR = 0.94, *p* = .144).

**Table 2:** Proportional hazards regressions predicting mortality from Log sIgA secretion rate, with and without adjustment for covariates.

		Model														
		1			2			3			4			5		
Cause of Death	Deaths	HR	95% CI	*p*	*HR*	95% CI	*p*	HR	95% CI	*p*	HR	95% CI	*p*	HR	95% CI	*p*
**All Causes**	300	0.81	0.73 to 0.91	< .001	0.85	0.76 to 0.95	.004	0.85	0.75 to 0.95	.004	0.86	0.76 to 0.96	.008	0.86	0.76 to 0.96	.008
**Cardiovascular Disease**	96	0.83	0.68 to 1.02	.070	0.86	0.71 to 1.06	.152	0.87	0.71 to 1.06	.167	0.89	0.72 to 1.09	.247	0.89	0.72 to 1.09	.247
**Respiratory Disease**	46	0.83	0.62 to 1.10	.197	0.88	0.66 to 1.17	.387	0.89	0.67 to 1.20	.454	0.92	0.69 to 1.22	.556	0.90	0.68 to 1.21	.495
**Cancer**	98	0.72	0.59 to 0.87	< .001	0.74	0.61 to 0.90	.002	0.74	0.61 to 0.90	.002	0.74	0.61 to 0.89	.002	0.74	0.61 to 0.90	.002
**Lung Cancer**	29	0.83	0.57 to 1.19	.301	0.93	0.65 to 1.32	.674	0.94	0.66 to 1.35	.752	0.93	0.65 to 1.33	.689	0.93	0.65 to 1.33	.689
**Other Cancer**	69	0.68	0.54 to 0.85	< .001	0.68	0.54 to 0.85	< .001	0.68	0.54 to 0.85	< .001	0.68	0.54 to 0.85	< .001	0.68	0.54 to 0.85	< .001
**Other causes**	60	0.98	0.75 to 1.28	.868	1.01	0.77 to 1.32	.956	1.01	0.77 to 1.32	.958	1.01	0.78 to 1.32	.922	1.01	0.78 to 1.32	0.917

Model1: adjusted for assay batch and sex. Model2: model1 + smoking status. Model3: model2 + Medications. Model4: model3 + Self-rated Health. Model5: model4 + manual social class.

Hazard ratios are per standard deviation of log sIgA secretion rate.

We also ran analogous analyses for sIgA concentration. The results revealed a similar pattern to sIgA secretion but with somewhat weaker effects (data not shown).

Table 3 shows the results for log sIgA and non-lung cancer stratified by sex. These are suggestive of a stronger association in men. However, in a model containing men and women, the sex by sIgA interaction was non-significant (*p* = .380) so any sex difference must be treated cautiously, albeit that the analysis has low power to detect an interaction.

**Table 3:** Proportional hazards regressions predicting non-lung cancer mortality from sIgA secretion rate, stratified by sex.

		Model														
		1			2			3			4			5		
	Deaths	HR	95% CI	*p*	HR	95% CI	*p*	HR	95% CI	*p*	HR	95% CI	*p*	HR	95% CI	*p*
**Men**	33	0.61	0.44 to 0.85	.004	0.61	0.44 to 0.86	.005	0.61	0.44 to 0.86	.005	0.61	0.44 to 0.86	.005	0.61	0.44 to 0.87	.005
**Women**	36	0.75	0.55 to 1.02	.066	0.74	0.54 to 1.02	.063	0.74	0.54 to 1.01	.057	0.73	0.53 to 1.00	.053	0.73	0.53 to 1.00	.052

Model1: adjusted for assay batch and sex. Model2: model1 + smoking status. Model3: model2 + Medications. Model4: model3 + Self-rated Health. Model5: model4 + manual social class.

Hazard ratios are per standard deviation of log sIgA secretion rate.

**Fig 1:**
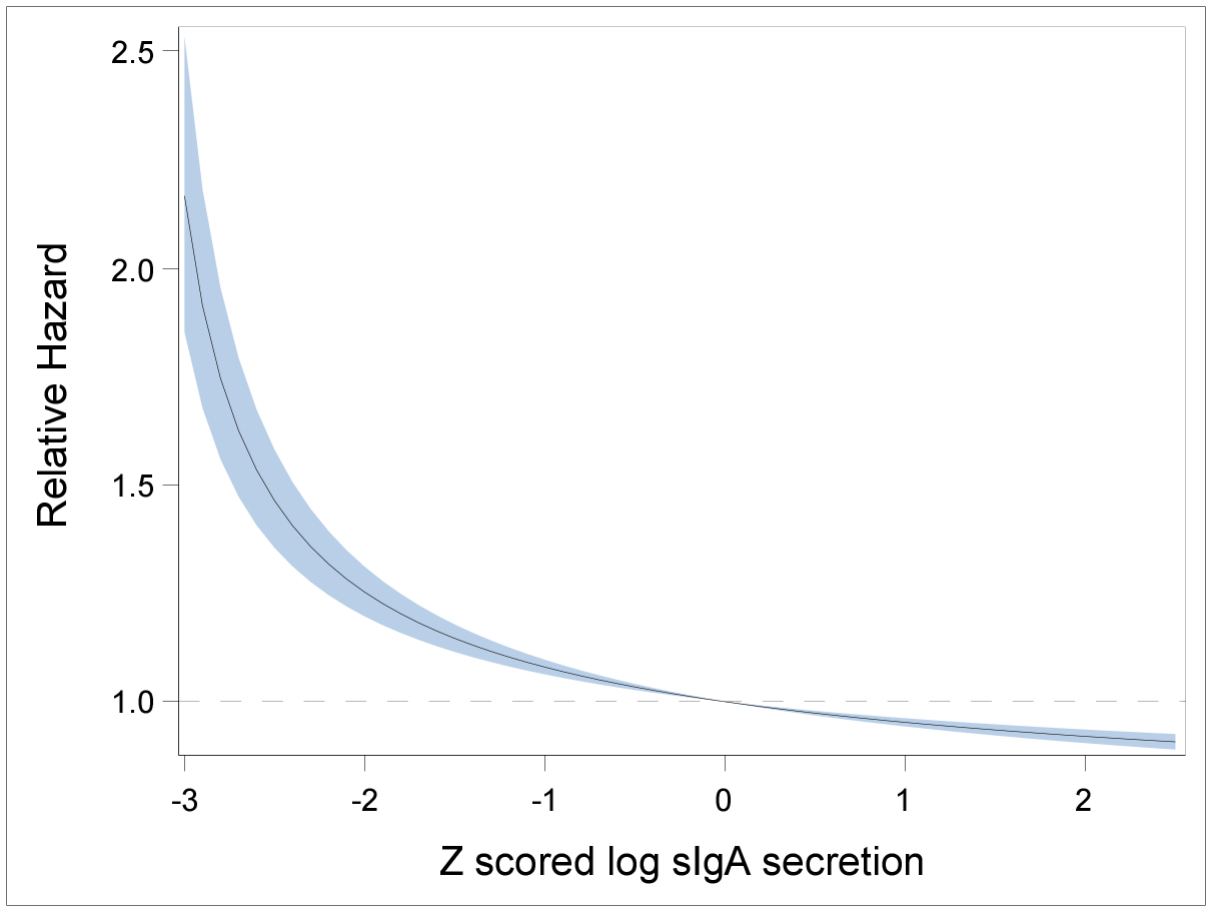
Relationship of (z scored) log sIgA secretion to respiratory mortality risk. **Fractional polynomial** model adjusted for assay batch and sex.

A further set of analyses employing polynomial terms in log sIgA secretion revealed a cubic relationship to mortality from respiratory diseases. The results of fitting this model are shown in Table 4. There was no evidence of non-linear effects for other causes or all causes taken together (results not shown). With only 46 deaths from respiratory diseases there is limited power to delineate with any precision the shape of the relationship. To provide an indicative result we have fitted a fractional polynomial in log secretion to the baseline model controlling for assay batch and sex. Fig 1 shows the relationship predicted from this model. This shows the mortality risk increasing steeply at two standard deviations below the mean, with no evidence of an increase in mortality risk at high levels of sIgA. Thus, the data provide no support for an increased mortality risk at high levels of sIgA whether for respiratory disease or any other causes.

**Table 4:** Results of model fitting cubic function of log sIgA secretion to respiratory disease mortality.

	Model														
	1			2			3			4			5		
Polynomial term	B	SE	*p*	B	SE	*p*	B	SE	*p*	B	SE	*p*	B	SE	*p*
**Linear**	0.40	0.24	.097	0.52	0.24	.031	0.47	0.24	.048	0.48	0.24	.045	0.43	0.23	.062
**Quadratic**	-0.00	0.14	.984	-0.04	0.14	.787	0.02	0.14	.905	0.01	0.14	.936	0.03	0.13	.807
**Cubic**	-0.13	0.06	.033	-0.15	0.06	.011	-0.12	0.06	.035	-0.12	0.06	.039	-0.11	0.05	.047

Model1: adjusted for assay batch and sex. Model2: model1 + smoking status. Model3: model2 + Medications. Model4: model3 + Self-rated Health. Model5: model4 + manual social class.

Hazard ratios are per standard deviation of log sIgA secretion rate.

In the present analysis we have observed that individuals with higher sIgA secretion rates have a decreased risk of all-cause mortality compared to individuals with lower secretion rates. This translates into a 19% decreased risk of mortality per standard deviation increase in log sIgA secretion rate. This direction of association is in line with the important role sIgA plays in defence against infections [3, 4], although there were not enough deaths by infectious disease in this cohort to analyse these separately.

Interestingly, of the separate main causes of death, cancer mortality was most strongly associated with sIgA, and, in particular, cancers other than lung cancer, suggesting that it was these latter deaths driving the overall link between low sIgA secretion rates and all-cause mortality. This is in line with a recent Swedish study of IgA deficiency whereby cancer deaths among those with IgA deficiency included higher proportions of prostate and colon cancers [21] although cancer mortality overall and for these subtypes was not *significantly* associated with IgA deficiency. In other studies, IgA deficiency has been significantly related to cancer risk [32–34]. Similarly, among immunodeficient cancer patients receiving bone marrow transplantation and developing infections, low levels of serum IgA related to mortality from cancer [35]. As immunodeficiency is heritable, it is possible that levels of sIgA in general are also partly heritable and this might explain why the association with mortality is not apparent for lung cancer, as lung cancer is primarily environmentally caused.

The mechanisms for the link between low sIgA and cancer mortality might be that low sIgA rates may reflect reduced immune surveillance at mucosal sites and in the glands that drain into them, and thus a higher risk of cancer emerging. Reduced immune surveillance in general would also underpin the observed associations between IgA deficiency and the increased risk of autoimmune [22, 36] conditions [32, 37, 38] in the Swedish study [21]. Nonetheless, it is difficult to directly compare serum IgA deficiency studies with those examining sIgA in saliva. Few studies have measured both or have focussed on populations with specific diseases or disease risk associated with low sIgA, such as HIV [39]. However, recently it has been observed that low levels of sIgA have been found among individuals with diabetes mellitus [17], and may be a mechanism of increased susceptibility to infections among diabetic patients, although others have found no significant differences between diabetics and controls [40]. Interestingly, it has also been noted that with ageing, serum levels of IgA increase [41] while migration of IgA producing B cells to the mucosa is impaired [42], resulting in both low sIgA and high serum IgA. Indeed, in some studies salivary and serum IgA are not correlated [40]. This makes it plausible that in older adults, both low sIgA and high serum IgA can be related to increased mortality. Some recent studies have shown higher serum IgA values associated with increased mortality risk [20, 41, 43]. However, in these cases the authors conclude that this is likely to be due to reverse causation whereby extant disease is responsible for elevated IgA levels. This latter observation taken together with the previous literature suggest several mechanisms by which low sIgA and high IgA might both relate to mortality; low sIgA might indicate impaired immunity with ageing, which in turn influences both infectious disease risk and cancer risk, whereas high serum IgA may indicate already underlying disease. Nevertheless, it should also be noted that in some instances, abnormally high sIgA levels indicate current acute oral infection [7], and inflammation [8].

The results here are also suggestive of a sex differences. When analysed separately by sex, the association with non-lung cancer mortality was stronger among men. However, this should be treated with caution as the interaction of sIgA with sex was not statistically significant. Interestingly, in the present study, males were characterised by higher sIgA levels, whereas, as indicated, showing a stronger association between sIgA and mortality. Previous studies have shown similar sex differences in sIgA [44], although, it should be conceded, others have reported no such differences [15, 21, 45]. Increased serum IgA levels in males have also been reported, along with a parallel increased risk of cancer deaths [41]. The stronger association for males between sIgA and mortality, presently observed, suggest that low sIgA may also be a risk factor for cancer mortality, even among males who have higher average levels, and may reflect the higher serum IgA but lower sIgA observed with ageing [41, 42]. All the models showing a negative association between sIgA and mortality were adjusted for sex.

That fewer significant associations emerged for sIgA concentration suggests that individual variation in salivary flow can confound the values for concentration, in contrast to secretion rate, and thus provide additional error of measurement, making the likelihood of significant and genuine associations less likely.

The advantages of the present study are that, in contrast with the work on immune deficiency, the present analysis is the first population study to link low levels of sIgA with cancer mortality where IgA levels were in the normal range rather than indicative of immunodeficiency or an existing clinical condition. The present study also extends earlier research by including a range of important indicators of comorbidity (self-rated health, medications) as well as smoking behaviour, whereas previous studies failed to control for such potential confounders. One limitation of the study concerns loss to follow-up and emigration from the UK. Although the study is routinely informed of emigration, these data may be incomplete if participants have emigrated without notification.

Although sIgA makes up only a small proportion of salivary proteins [46], it is the most prolific antibody in saliva [47] and its negative association with infection risk [17] means that it is not entirely surprising that reduced sIgA secretion rates are associated with all-cause mortality risk in the present study, although a limitation of this analysis is the lack of sufficient numbers to examine infectious disease mortality effectively. However, these associations with respiratory infections do not explain the observed links with non-lung cancers and respiratory disease mortality. Further, future longitudinal research would benefit from tracking cancer and respiratory disease morbidity as well as mortality in order to better understand the mechanisms and pathways underlying the present associations. A comparison of sIgA and serum IgA in the context of specific mortality risk would also be informative in terms of the comparability of the prognostic value of these indicators.

## Conclusions

In conclusion, in the present analysis, we observed a decreased risk of 19 year all-cause mortality, specifically cancer mortality, among older adults with higher secretion rates of salivary IgA. This withstood adjustment for potential confounding variables including sex, assay batch, household occupational group, smoking, medication usage, and self-reported health. This is the first study to show this specific link between sIgA and mortality, and suggests that salivary IgA secretion rate could serve as an easily measurable marker of general mortality risk and particularly cancer-specific risk.
